# Integrated multi-trophic aquaculture with sugar kelp and oysters in a shallow coastal salt pond and open estuary site

**DOI:** 10.3389/faquc.2023.1147524

**Published:** 2023-05-09

**Authors:** Lindsay A. Green-Gavrielidis, Carol S. Thornber, Autumn Oczkowski

**Affiliations:** 1 Department of Natural Resources Science, University of Rhode Island, Kingston, RI, United States; 2 Atlantic Ecology Division, United States Environmental Protection Agency (US EPA), Narragansett, RI, United States

**Keywords:** kelp farming, biomass yield, nutrient extraction, sustainable aquaculture, oyster aquaculture

## Abstract

Sustainable aquaculture includes the aquaculture of non-fed crops that provide ecosystem services including nutrient extraction and water quality improvement. While shellfish are the most farmed sustainable aquaculture crops in the USA, shellfish farmers in the northeastern US have an interest in diversifying their crops and incorporating seaweeds into their farms. In this study, we worked with oyster farmers to investigate the potential for farming sugar kelp, *Saccharina latissima*, across different environmental regimes in coastal Rhode Island USA. Kelp seed spools were outplanted at two time points in the fall/winter of 2017 and 2018 at four sites and cultivated until harvest the following spring. Kelp performance (length, width, yield), tissue content, and nutrient extraction were determined for each line in each year; oyster growth was also measured monthly for one year at each site. We found that kelp could successfully grow in both shallow coastal lagoons and estuarine sites, although the timing of planting and placement of sites was important. Lines that were planted earlier (as soon as water temperatures<15°C) grew longer and yielded more biomass at harvest; overall, kelp blade yield ranged from 0.36 ± 0.01 to 11.26 ± 2.18 kg/m long line. We report little variation in the tissue quality (C:N) of kelp among sites, but differences in biomass production led to differences in nutrient extraction, which ranged from 0.28 ± 0.04 to 16.35 ± 4.26 g nitrogen/m long line and 8.93 ± 0.35 to 286.30 ± 74.66 g carbon/m long line. We found extensive variability in kelp growth within and between lines and between years, suggesting that crop consistency is a challenge for kelp farmers in the region. Our results suggest that, as there is a lower barrier in terms of permitting (versus starting a new aquaculture farm), it may be a worthwhile investment to add sugar kelp to existing oyster farms, provided they have suitable conditions. At current market rates of US$0.88-$3.30 per kg, farmers in southern New England have the potential to earn US$2,229 per 60 m longline. While seaweed aquaculture is growing, considerable barriers still exist that prevent wide-scale kelp aquaculture adoption by existing aquafarmers.

## 1 Introduction

Sustainable aquaculture focuses on the cultivation of crops, typically non-fed, that provide protein while optimizing environmental benefits. Integrated multi-trophic aquaculture (IMTA) is a type of sustainable aquaculture that is widely recognized as having significant environmental and economic benefits across local, regional, and global scales. There are documented benefits of shellfish on seaweed aquaculture (e.g., Marinho et al., 2015; Hargrave et al., 2022), demonstrating that kelp grown near mussels was significantly longer and more productive with lower epiphyte load (Hargrave et al., 2022) and a decrease in dissolved nutrients (Marinho et al., 2015). Similarly, the presence of cultivated seaweeds can reduce the accumulation of saxotoxins in bivalves (Sylvers and Gobler, 2021). Jiang et al. (2020) found that cultivation of the kelp *Saccharina japonica* (Areschoug) C.E. Lane, C. Mayes, Druehl & G.W. Saunders reduced dissolved inorganic nutrient concentrations, reduced suspended solids, and enhanced phytoplankton abundance and chlorophyll a. While over 99% of seaweed cultivation occurs in Asian and Pacific Rim countries, the United States has 3.4% of the market share (valued at US$55.9 million) for importing seaweeds for human consumption (Piconi et al., 2020), representing an underutilized opportunity for potential seaweed cultivation along its shores (Kim et al., 2019).

The sugar kelp (kombu) *S. latissima* (L.) C.E. Lane, C. Mayes, Druehl, & G.W. Saunders has a wide distribution in temperate to polar waters, including the northeastern and northwestern Atlantic Ocean, as well as the northeast Pacific Ocean, Japan, and the Arctic Ocean (Guiry & Guiry, 2022). While there is a long history of kelp cultivation in Asian and Pacific Rim countries, particularly for *S. japonica* and *Undaria pinnatifida* (Harvey) Suringar (Gao et al., 2013; Hwang et al., 2019; Hu et al., 2021), there has been recent interest in cultivation of *S. latissima* in emerging macroalgal markets in Europe and the United States (e.g., Kim et al., 2019; Matsson et al., 2019; Forbord et al., 2020). This species is an ideal candidate for aquaculture due to its ease of cultivation, annual life cycle when cultivated, and rapid growth rate (Marinho et al., 2015; Fossberg et al., 2018; Broch et al., 2019), including along the U.S. northern Atlantic coastline (Augyte et al., 2017; Kim et al., 2019).

*S. latissima* is primarily raised for direct human consumption, although other farmed kelps worldwide also contribute significantly to markets for food additives, animal feed, and pharmaceutical products (Peteiro et al., 2016; Purcell-Meyerink et al., 2021). There is considerable interest in raising the protein content in farmed kelp for animal feed uses (Aasen et al., 2022). In 2016, its estimated economic value in Europe alone was 40–49 euros/kg dry mass (Peteiro et al., 2016). In the United States in 2019, all farmed macroalgae for human consumption yielded 55,000–60,000 estimated dry pounds; at least 85% of this was from kelp species (Piconi et al., 2020). This market is growing rapidly, and there was nearly a doubling in farmed kelp biomass in Maine from 2019 to 2020 (28,000 dry pounds to 50,000 dry pounds; Maine Department of Marine Resources, 2021).

In New England USA, there is considerable interest by oyster farmers with existing water leases in cultivating multiple species within a single leased area, to maximize profits while minimizing costs (Coastal Enterprises, 2018). While oysters exhibit maximum growth rates during the summer months, kelps have colder optimum temperatures (Bolton and Lüning, 1982) and grow during the winter months in New England (Augyte et al., 2017). However, the potential for maximum kelp growth can vary across small spatial scales, as kelp farmers may have abutting leases that vary in water depth, clarity, nutrient content, and/or water velocity (Matsson et al., 2019; Forbord et al., 2020; Visch et al., 2020; Stekoll et al., 2021). The physical requirements for optimal kelp growth may differ from those required for optimal cultivation of shellfish such as oysters, mussels, and clams, which may cause conflicts among the siting of leased areas, as well as for other water uses (Knapp and Rubino, 2016).

In this study, we worked with local oyster farmers to investigate the potential for successfully farming *S. latissima* across different environmental regimes (salt pond, open estuary) in established oyster farms in coastal Rhode Island USA. We assessed the impacts of planting timing and farm location on kelp growth, yield, tissue content, and nutrient extraction. Additionally, we quantified the annual growth cycle of oysters on each farm. We use our findings to assess the impacts of kelp farming on local nutrient extraction, as well as to determine the suitability of kelp farming in this region.

## 2 Materials and methods

### 2.1 Spore release and nursery cultivation

Reproductive *S. latissima* was collected *via* SCUBA diving at Ft. Wetherill, RI, USA (41.478007, −71.360620) in late summer and fall of 2017 and 2018 and placed on ice for transportation to the lab. This site has an expansive kelp population and recent population genetic analyses have reported that within site diversity of kelp in southern New England is higher than between sites (Mao et al., 2020). In the lab, ten individuals were selected at random, measured, and processed for spore release each time, following the protocols of Redmond et al. (2014) and Flavin et al. (2013). Briefly, reproductive sorus tissue was excised, cleaned to remove epiphytes or other contaminants, and placed in damp, paper towels in the refrigerator overnight. The following day, all sori were placed in sterile seawater (10°C) and monitored for zoospore release. After zoospore release occurred, spore density was determined using a hemocytometer and the zoospore solution was filtered through sterile cheesecloth. The volume of the zoospore solution was adjusted to a density of 7,500 spores/mL and used to inoculate seed spools (5 × 36 cm PVC pipes wrapped with nylon twine).

Seed spools were maintained in the dark at 10°C overnight before being transferred to temperature-controlled aquaria. Seed spools were maintained at 10°C and provided with 20–30 μmol photons m^−2^ s^−1^ on a 12:12 Light: Dark photoperiod and provided with ½ strength Provasoli’s Enriched Seawater in aquaria; germanium dioxide was added to aquaria to discourage diatom growth (Redmond et al., 2014). Spools were transferred to clean aquaria with fresh nutrients weekly and light levels were adjusted according to Flavin et al. (2013) to encourage gametophyte development, reproduction, and sporophyte development. After approximately 6–8 weeks, kelp sporophytes were ready to be planted at farm sites. Field cultivation occurred over two different growing seasons, Year 1 (2017–2018) and Year 2 (2018–2019). In each growing season, we collected reproductive wild *S. latissima* twice and inoculated seed spools each time.

### 2.2 Farm sites and planting kelp

**FIGURE 1:**
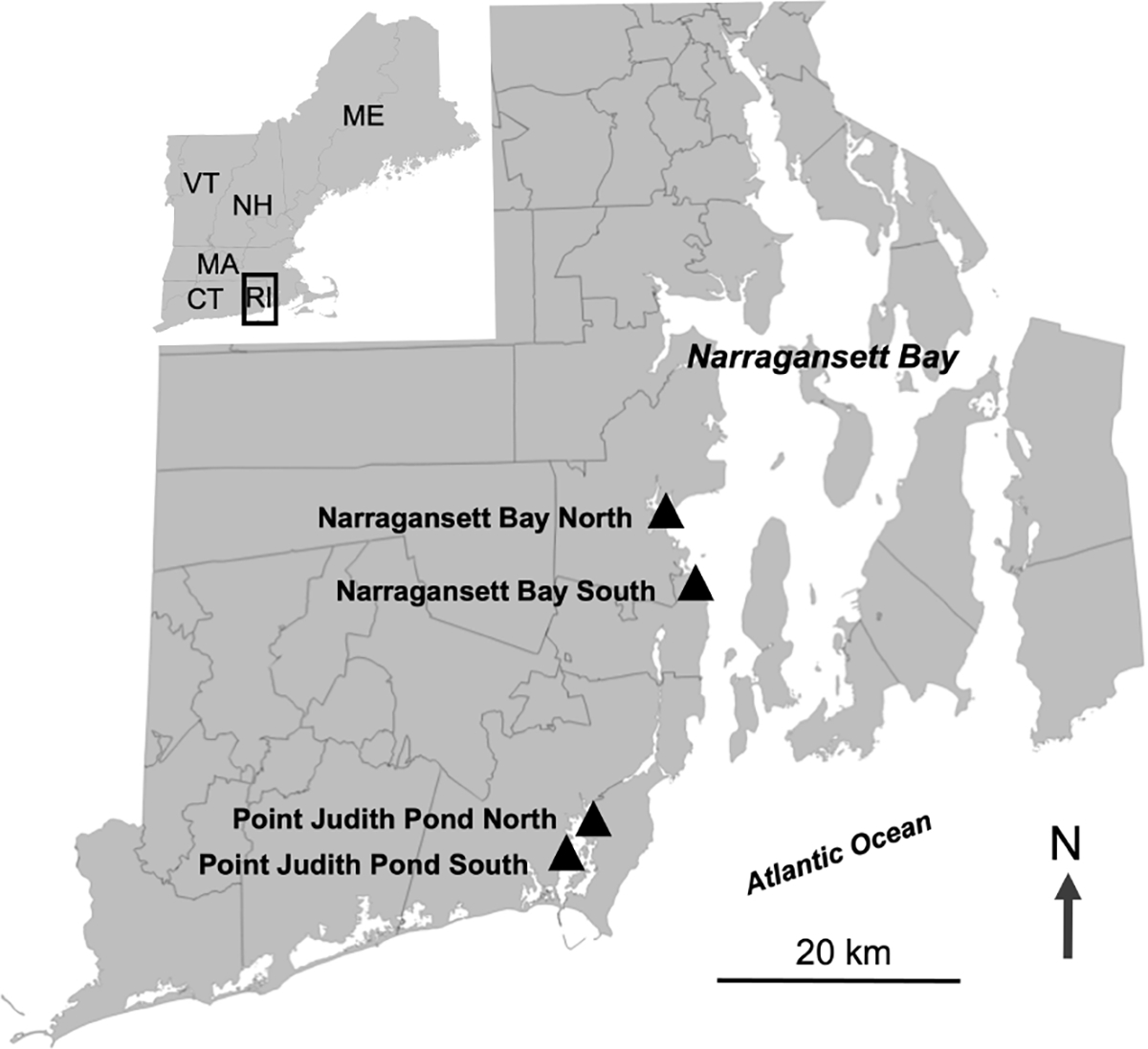
Map showing the four sites where sugar kelp was planted on existing oyster farms. Two sites were located in the West Passage of Narragansett Bay and two sites were located in Point Judith Pond.

We established partnerships with commercial oyster farmers to add kelp to four existing aquaculture lease sites in Rhode Island. One of the primary aims of this study was to determine if kelp could be successfully integrated into shallow water oyster farms since a large amount of oyster aquaculture happens in coastal lagoons (also called salt ponds) in Rhode Island. Therefore, we had two sites in Narragansett Bay, Narragansett Bay North (Narr Bay N) and Narragansett Bay South (Narr Bay S), and two sites in a coastal lagoon, Point Judith Pond North (Pt. Judith N) and Point Judith Pond South (Pt. Judith S; Figure 1). The Narr Bay N site was located on the seaward side of a jetty wall in the west passage of Narragansett Bay at the entrance of a marina and had an average depth of 3–4 m at low tide. Narr Bay S was also located in the western passage of Narragansett Bay, approximately 4 km south of Narr Bay N, with an average depth of 8–10 m at low tide. Pt. Judith N and S were located less than 1 km apart in Point Judith Pond, a tidally influenced coastal lagoon between the towns of Narragansett and South Kingstown, RI. The average depth of Pt. Judith N was 2–3 m at low tide, while the average depth of Pt. Judith S was 3–4 m at low tide. Oyster cultivation at Narr Bay N, Narr Bay S, and Pt. Judith N was bottom rack and bag, while cultivation at Pt. Judith S was uncaged bottom culture. Floating oyster racks were added to the Narr Bay S site in Year 2 adjacent to the kelp longlines (but not over them).

**FIGURE 2:**
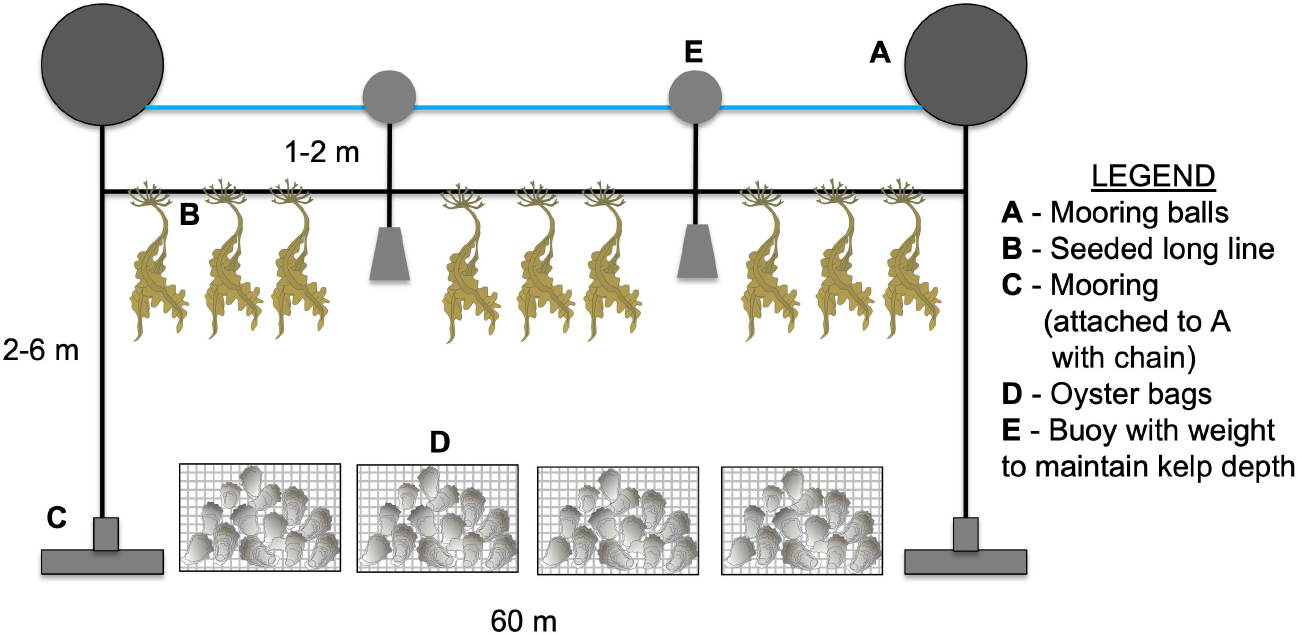
Long line design for oyster and sugar kelp IMTA. Two long lines (60 m) were deployed at each site in Year 1 and Year 2 directly adjacent to the bottom oyster bags (Narr Bay N, Narr Bay S, Pt. Judith N) or open bottom culture of oysters (Pt. Judith S).

At each site, we planted two 60 m lines of seed string at different time periods to determine the ideal planting time in each growing season (Year 1 and Year 2). Planting occurred in the fall after the water temperature fell below 15°C, or when the seed spools from our nursery were ready (Table S1). During planting, twine from seed spools were wrapped around a larger long line and deployed 1 m (Point Judith Pond) or 2 m (Narragansett Bay) below the water surface (Figure 2). Buoys were attached to the long line at periodic intervals to maintain the kelp at the desired depth.

### 2.3 Environmental data

Temperature loggers (HOBO Water Temperature Pro v2 Data Logger, Onset Computer Corporation, Bourne, MA, USA) were attached to kelp lines at each site to collect continuous data during the growth season. In addition, each farm site was visited monthly during the kelp cultivation season (weather dependent). During each farm visit, surface water samples were collected for dissolved nutrient analysis using acid-washed bottles; we rinsed the bottle three times in surface water before final collection. Water samples were filtered and frozen at −80°C until analysis. In Year 1, nitrate, nitrite, ammonium, and phosphate concentrations were measured using a LACHAT Flow Injection Autoanalyzer (LACHAT, 2008). In Year 2, nitrate, nitrite, ammonium, and phosphate concentrations were determined using an Astoria Pacific Model 303A Segmented Continuous Flow Autoanalyzer (Astoria-Pacific Inc, Clackamas, OR; Eaton et al., 1998). Temperature, conductivity, salinity, pH, chlorophyll a, and dissolved oxygen were also measured using a YSI 6560 Sonde lowered to the depth of the kelp line.

### 2.4 Kelp performance and tissue content

To determine kelp performance, we collected kelp samples (n=8–15) haphazardly from each line during farm visits. Samples were placed on ice and brought back to the laboratory where maximum blade length, maximum blade width, stipe length, and holdfast length, where applicable, were recorded. A different subsample of blades (excluding the holdfast and stipe) was then processed for percent carbon and nitrogen determination. Briefly, blades were dried to a constant mass and ground into a powder in a mortar and pestle. Subsamples of ground tissue (2 – 3 mg) were encapsulated in tins (3.5 × 5 mm) and analyzed at the United States Environmental Protection Agency, Atlantic Division Laboratory in Narragansett, RI, USA. Carbon, nitrogen, δ^15^N, and δ^13^C content of tissue samples was measured using an Isoprime 100 Isotope Ratio Mass Spectrometer interfaced with a Micro Vario Elemental Analyzer (Elementar Americas, Mt. Laurel, New Jersey, USA).

Kelp blade length (mean ± SD) at all four sites in Year 1 and Year 2 are reported in Venolia et al. (2020). Venolia et al. (2020) used these data to calibrate a dynamic energy budget model for *S. latissima* and to test how well the final model accurately predicted kelp growth over time by comparing model outputs to the field collected data. In this study, we were interested in determining differences in blade length and blade width among sites and months using 2-way analysis of variance (ANOVA). We therefore conducted separate analyses for each line (Line 1 and Line 2) in each year (Year 1 and Year 2). We used a Bonferroni correction to account for two different analyses (length and width) on the same samples with a threshold p-value of 0.025. We also analyzed differences in δ^15^N, δ^13^C, %N, %C, and C:N among sites and months using 2-way ANOVAs; separate analyses were conducted for each line in both years. To account for the fact that we conducted five different statistical analyses with data from the same samples, we used a standard Bonferroni correction with a p-value threshold of 0.01 for these analyses. Prior to all analyses, data were tested for normal distribution and heterogeneity of variances and transformations were performed, where necessary, to ensure variances were equal. In some instances, data did not meet the assumption of normality even after transformation, however ANOVA has been shown to be robust to deviation from normality when experimental designs are balanced and sample sizes are reasonable (Underwood, 1997). When data did not meet the assumption of equal variances after transformation, we used rank transformation (Conover and Iman, 1981) and subsequently conducted the ANOVA analysis. Due to logistical issues associated with conducting field work, there were some missing data points which required data to be excluded from some analyses. Details on which data were excluded from which analyses and how data were transformed are provided in Table S2. All ANOVA analyses were conducted in JMP Pro 15.2 (SAS Institute Inc, Cary, NC, USA).

### 2.5 Oyster performance

To determine oyster performance, a subset of oysters (n = 30) from each site (n = 4) were individually tagged with mini passive integrated transponder (PIT) tags (HPT8 PIT Tags, Biomark, Boise, ID) embedded in marine epoxy. PIT tags were measured with a transponder that gave a unique number to allow us to track the growth rate of each individual oyster. For each oyster, shell height and shell width were measured using calipers to the closest 0.1 mm beginning in May 2018; oysters were then deployed back onto the farms with a water temperature logger (Hobo Water Temperature Pro v2, Hobo Onset, Bourne, MA) programmed to take a measurement every 15 minutes. Oysters were measured monthly from May 2018 until May 2019 (except in November 2018, January 2019, and April 2019 due to weather); any death of individual tagged oysters was noted each month. This study was exempt from animal welfare approval since it did not include any vertebrate animals; the University of Rhode Island is fully compliant with all animal welfare regulations.

To assess differences in oyster growth across sites, we conducted split-plot ANOVAs (JMP v15.2) for oyster height growth rate and oyster width growth rate with Site as the main plot and Time as the sub-plot. Growth rates were calculated as height (or width) growth (mm/day) for each time period (monthly or bimonthly, depending upon the frequency of sampling). Thus, while we had ten data points (months) for each oyster’s height and width, we had nine data points for each oyster’s height growth/day and width growth/day. These data did not meet the assumption of equal variances after transformation; thus, we used rank transformation (Conover and Iman, 1981) and subsequently conducted ANOVA analysis. A standard Bonferroni correction was used to account for multiple (n = 2) analyses from the same oysters with a threshold p-value of 0.025. In addition, we conducted a chi-square analysis to assess differences in survival across the four sites (Excel v.16.59).

### 2.6 Kelp yield and nutrient extraction

At the time of harvest, 10 cm sections of longline (n = 3 from each line) were collected fully intact and transported to the laboratory on ice to determine yield. In the laboratory, kelp was rinsed to remove epiphytes and for each 10 cm section, blades, stipes, and holdfasts were manually separated. Tissue was spun in a salad spinner to remove excess water and the fresh mass was obtained and used to calculate biomass yield as kg kelp per m of longline. For each line, we used the following equation from Kim et al. (2014) to calculate the carbon and nitrogen extraction of each line: $\text{N}\;\left(\text{or}\;\text{C}\right)\;\text{extraction}\;=\frac{\text{gFW}\;\text{produced}}{\text{m}}*\frac{\text{g}\;\text{DW}}{\text{g}\;\text{FW}}*\frac{\text{g}\;\text{N}\;\left(\text{or}\;\text{C}\right)}{\text{g}\;\text{DW}}$ using the blade mass (g fresh weight, FW) and average carbon and nitrogen content measured at harvest. We determined the relationship between kelp dry weight (DW) and FW by taking 35 individual blades across lines and sites, measuring their fresh weight and then drying to a constant mass and measuring their DW. Our calculated DW: FW ranged from 0.04 – 0.13 with an average of 0.1 ± 0.004); 0.1 was used as the standard DW: FW in the above calculation.

### 2.7 Gross revenue

We used the average blade biomass (kg per m) at harvest produced on Line 1 at each of the sites in each of the years to calculate the gross revenue that could be earned by cultivating kelp in Rhode Island. Blade biomass (kg per m) was multiplied by 60 m to represent the standard size of a single longline. The total biomass produced in 60 m was then multiplied by lower end market rates of $0.88 per kg (Piconi et al., 2020) and higher end market rates of $3.30 per kg (Engle et al., 2018).

## 3 Results

### 3.1 Environmental data

There was considerable variability among sites and over time in the inorganic phosphate (PO_4_-P) and total inorganic nitrogen (DIN; NO_3_, NO_2_ and NH^+^_4_) measured near kelp lines (Tables S3–S6). DIN was generally lower at the Narragansett Bay sites compared to the Pt. Judith Pond sites; PO_4_-P ranged from not detectable to 1.07 μM, while DIN ranged from not detectable to 12.15 μM during the study period. There was a general decrease in DIN and PO_4_-P from winter to spring. The Narragansett Bay sites tended to warm more quickly in the springtime, while the Pt. Judith Pond sites experienced higher peaks in chlorophyll a. Temperatures logged on the kelp lines during the study period ranged from −0.80°C to 14.5°C, while chlorophyll a ranged from 1.4 to 58.7 μg/L. No clear differences in salinity or pH were identified among the sites; salinity ranged from 26.52 to 31 while pH ranged from 7.6 to 8.36 (Tables S3–S6).

### 3.2 Kelp performance

#### 3.2.1 Blade length

Kelp blade length (mean final length 64.10 cm ±7.14) at all four sites in Year 1 and Year 2 are reported in (Figure 7 and Table 4 in Venolia et al., 2020); here we report the results of new analyses examining differences in blade length among sites and over time. In both years, kelp grew at all sites and the final kelp length on Line 1, which was planted earlier, was on average 48.31% longer than on Line 2. Kelp blade length on both lines during each year varied significantly among sites, though this was dependent on time (Table 1). Blade length increased significantly over time at nearly all sites and lines; the final blade length for Line 1 was longest at Narr Bay S (Year 1) and Pt. Judith Pond N (Year 2) and shortest at Narr Bay N (both years), and for Line 2 was longest at Pt. Judith S (both years) and shortest at Narr Bay N (Year 1) and Narr Bay S (Year 2). The rate of increase slowed between March and April at all sites in Year 1 except Narr Bay N for Line 1; Pt. Judith S had a significant decline in blade length between April and May in Year 2, Line 2 (see Venolia et al., 2020 for original data).

#### 3.2.2 Blade width

**FIGURE 3:**
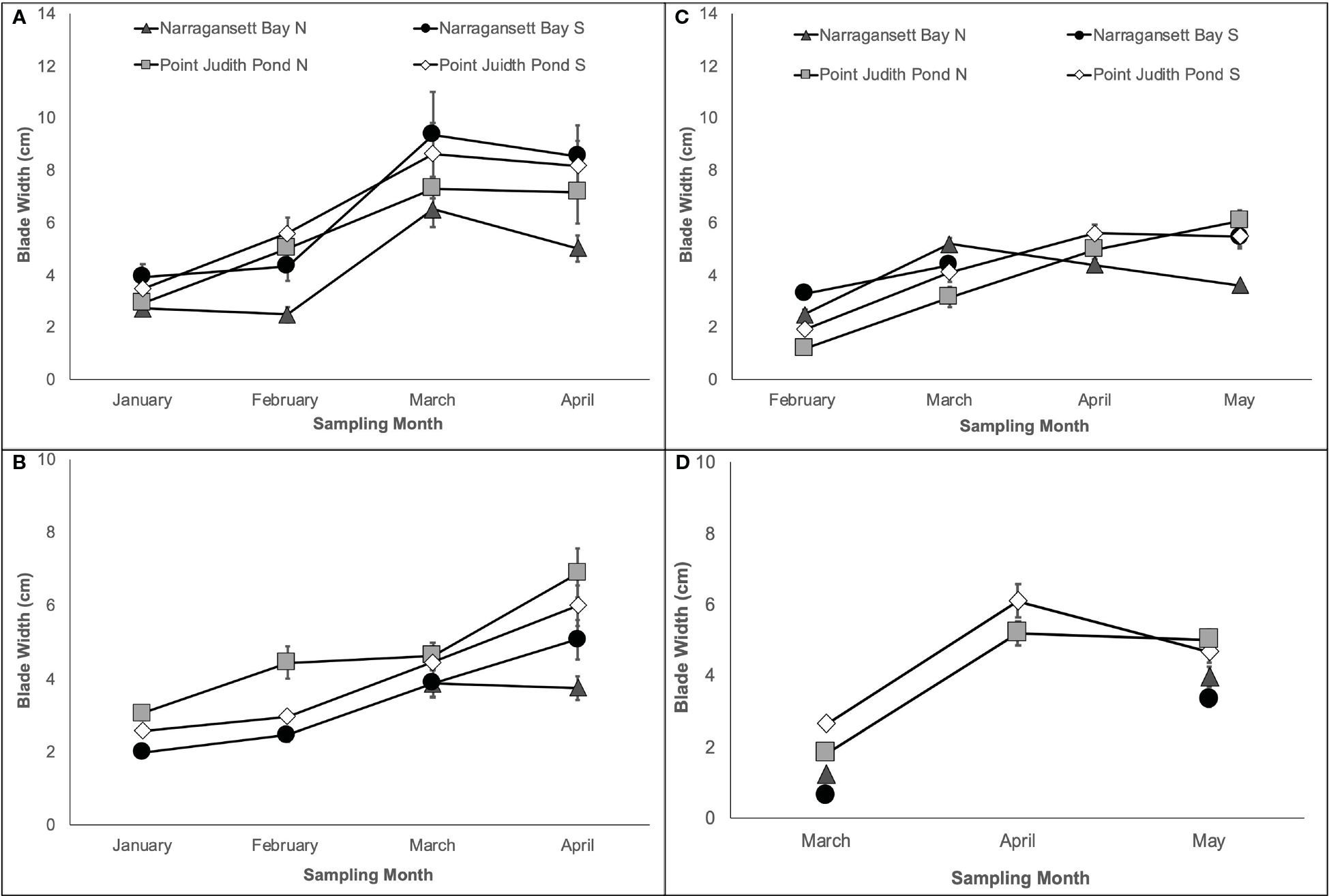
Kelp blade width from Year 1 showing Line 1 **(A)** planted in November and Line 2 **(B)** planted in December and Year 2 showing Line 1 **(C)** planted in December and Line 2 **(D)** planted in January.

In both years, the final kelp width was on average 29.34% larger on Line 1, which was planted earlier, than on Line 2. As with blade length, final blade width was largest at Pt. Judith N and smallest at Narr Bay N in most cases. Kelp blade width on each line during Year 1 varied significantly among sites and over time (Table 1; Figures 3A, B). On Line 1, blades in April (7.34 cm ± 0.53) were significantly wider than blades in February (4.36 cm ± 0.31) and January (3.27 cm ± 0.21; p< 0.0001; Figure 3A). Final blade width of Line 1 was largest at Narr Bay S (8.54 cm ± 1.17) and smallest at Narr Bay N (5.01 cm ± 0.52; Figure 3A). On Line 2, blades were wider at Pt. Judith N than at Pt. Judith S and Narr Bay S (p< 0.0001; Figure 3B); Narr Bay N was excluded from this analysis.

In Year 2, blade width increased on Line 1 over time, but the pattern differed by site Table 1; Figure 3C). Final blade width of Line 1 was largest at Pt. Judith N (6.07 cm ± 0.41) and smallest at Narr Bay N (3.6 cm ± 0.14; Figure 3C). Blade width of kelp on Line 2 increased over time from March to May, with patterns differing among sites (Table 1; Figure 3D). Final blade width of Line 2 was largest at Pt. Judith N (5.02 cm ± 0.3) and smallest at Narr Bay S (3.98 cm ± 0.28; Figure 3D).

### 3.3 Oyster performance

#### 3.3.1 Oyster growth

**FIGURE 4:**
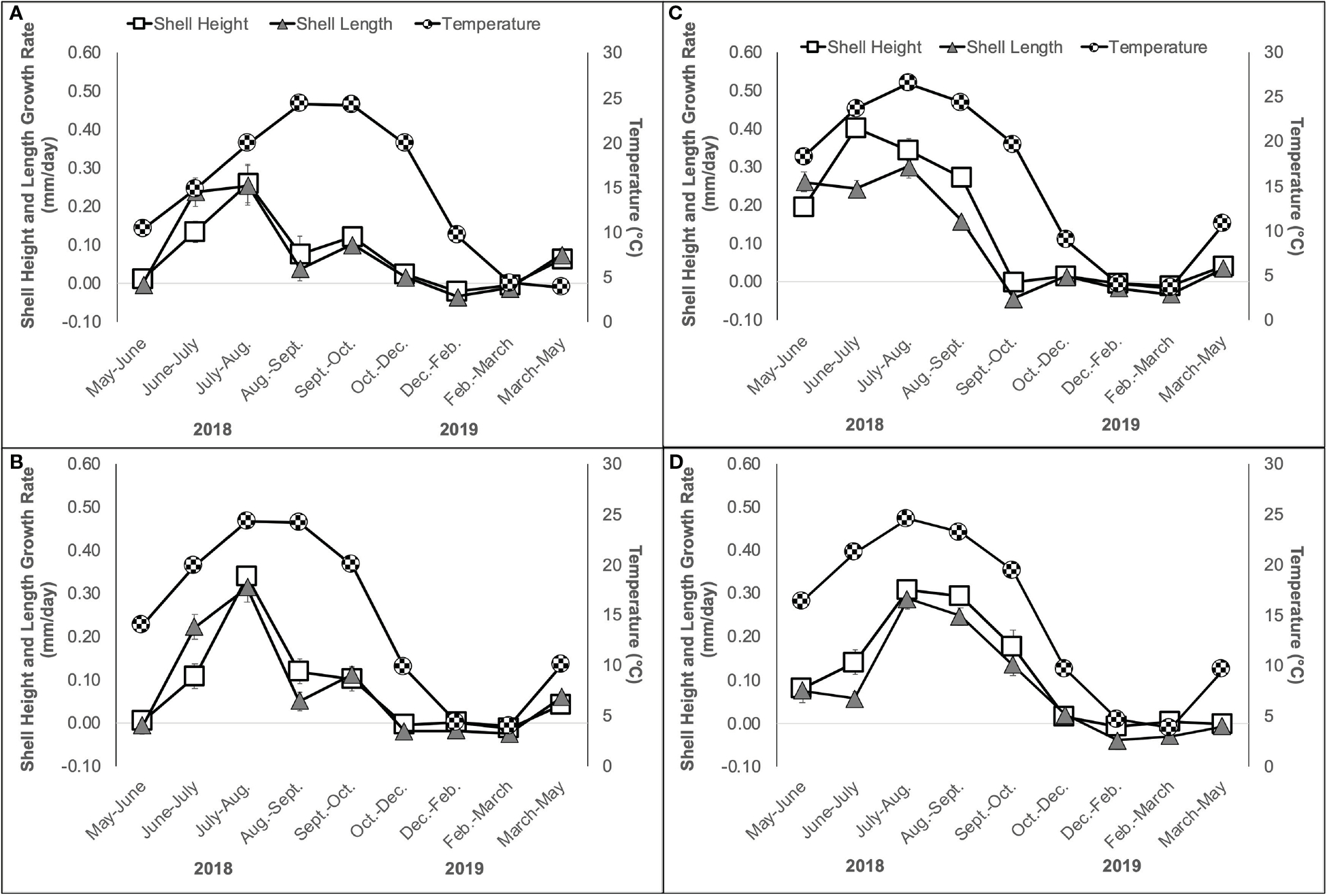
Oyster shell height (mm/day) and shell length (mm/day) growth rate and temperature (C, second y-axis) ± SE over a 12-month period from May 2018 to May 2019 at Narr Bay N **(A)**, Narr Bay S **(B)**, Pt. Judith N **(C)** and Pt. Judith S **(D)**.

Oyster shell height and length growth rates (mm/day) varied significantly among sites, but this effect was dependent on time in both cases (Table 1; Figures 4A–D). The growth rates of shell height and length were similar within a site, with the peak growth occurring in July-August at Narr Bay N (Figure 4A) and Narr Bay S (Figure 4B), from June-July at Pt. Judith N (Figure 4C), and from July-September at Pt. Judith S (Figure 4D). Temperature was significantly positively correlated with both oyster shell height and length growth rates at all sites (Pearson’s correlation r = 0.7392 – 0.9213), except Narr Bay N.

#### 3.3.2 Oyster survival

We found marginally significant differences (χ^2 =^ 7.66, df = 3, p = 0.05) in oyster survival among the sites from May 2018 – May 2019. Survival ranged from 52% (Narr Bay S), 68% (Pt. Judith S), 76% (Narr Bay N), to 83% (Pt. Judith N).

### 3.4 Tissue content

In all cases except for Year 1 Line 1, the kelp grown in Narragansett Bay had a higher δ^15^N than kelp from Point Judith Pond. The δ^15^N of kelp blades varied significantly by site in all cases (Table 1; Figures S1A–D). In Year 1, kelp blades from Line 1 from Narr Bay S had a significantly higher δ^15^N (10.15‰ ± 0.36) than Pt. Judith N (6.47‰ ± 0.5; p = 0.006), but they were not different from Narr Bay N (8.55‰ ± 0.26) and Pt. Judith S (6.79‰ ± 0.24; Figure S1A). In Year 2, kelp δ^15^N also increased over time, with higher δ^15^N in Line 1 in May (9.31‰ ± 0.41) compared to February (7.25‰ ± 0.31; p< 0.0001) and March (7.63‰ ± 0.35; p = 0.0001; Table 1; Figure S1C). The δ^15^N of kelp from Line 2 increased over time at the Narr Bay Sites only (Table 1; Figure S1D).

The mean δ^13^C of kelp blades ranged from −25.33‰ ± 0.36 to −17.33‰ ± 0.15 (Figure S2). The δ^13^C of kelp blades varied by site and month in all cases except Year 2 Line 2 (Table 1; Figure S2A–C). During Year 2, the δ^13^C of blades on Line 2 varied by site (Table 1; Figure S2D) with δ^13^C in blades from Narr Bay S significantly lower (−23.70‰ ± 0.24) than blades grown at Pt. Judith N (−21.07‰ ± 0.44; p< 0.0001) and Pt. Judith S (−21.83‰ ± 0.14; p< 0.0001).

The mean percent of kelp nitrogen ranged from 0.51% ± 0.02 to 3.53% ± 1.18 (Figure S3), and %N at Pt. Judith N was higher than Pt. Judith S and Narr Bay S. Tissue %N varied by site and month on kelp from Year 1 Line 1 (Figure S3A) and Year 2 Line 1 (Table 1; Figure S3C), with %N generally decreasing over time. Kelp from Year 1 Line 2 showed a different pattern, varying by site and among months with no significant interaction (Table 1; Figure S3B). In Year 2, kelp %N on Line 2 remained constant over time but showed similar differences among sites (Table 1; Figure S3D).

The mean percent carbon of kelp ranged from 20.39% ± 0.80 to 31.77% ± 6.14. In Year 1, there was no significant effect of either site or month on the %C on Line 1 (Figure S4A) or Line 2 (Table 1; Figure S4B). In Year 2, there was a significant difference in the %C of kelp among sites for Line 1, but not Line 2 (Table 1; Figure S4C, D). On Line 1 in Year 2, the %C of kelp blades grown at Narr Bay S was significantly higher than kelp grown at Pt. Judith N and Pt. Judith S.

**FIGURE 5:**
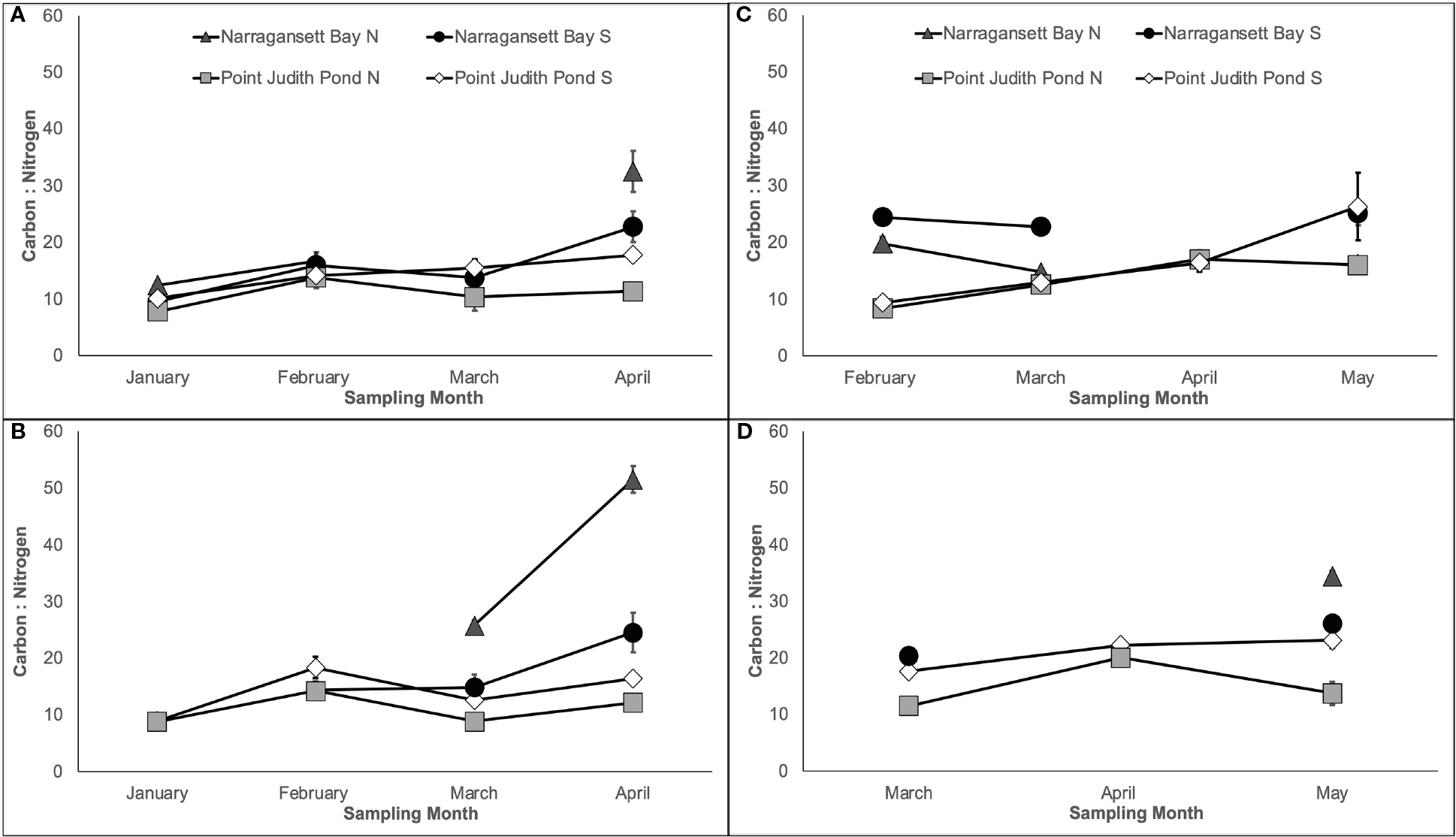
Percent carbon: percent nitrogen of kelp blades grown from Year 1 showing Line 1 **(A)** planted in November and Line 2 **(B)** planted in December and Year 2 showing Line 1 **(C)** planted in December] and Line 2 **(D)** planted in January.

The mean C:N of kelp ranged from 7.79 ± 0.15 to 51.53 ± 2.36 and generally increased over time, although the magnitude was site dependent (Figure 5). The C:N varied significantly among sites; this was dependent on the month in Year 1 on both lines (Figures 5A, B) and in Year 2 on Line 1 (Table 1; Figure 5C). The C:N of kelp in Year 2 on Line 2 varied by site and month (Table 1; Figure 5D) and kelp grown at Narr Bay S (23.19 ± 1.17) was significantly higher than Pt. Judith N (12.61 ± 1.03; p< 0.0001) and Pt. Judith S (20.06 ± 1.19; p< 0.0001).

### 3.5 Kelp biomass yield

**FIGURE 6:**
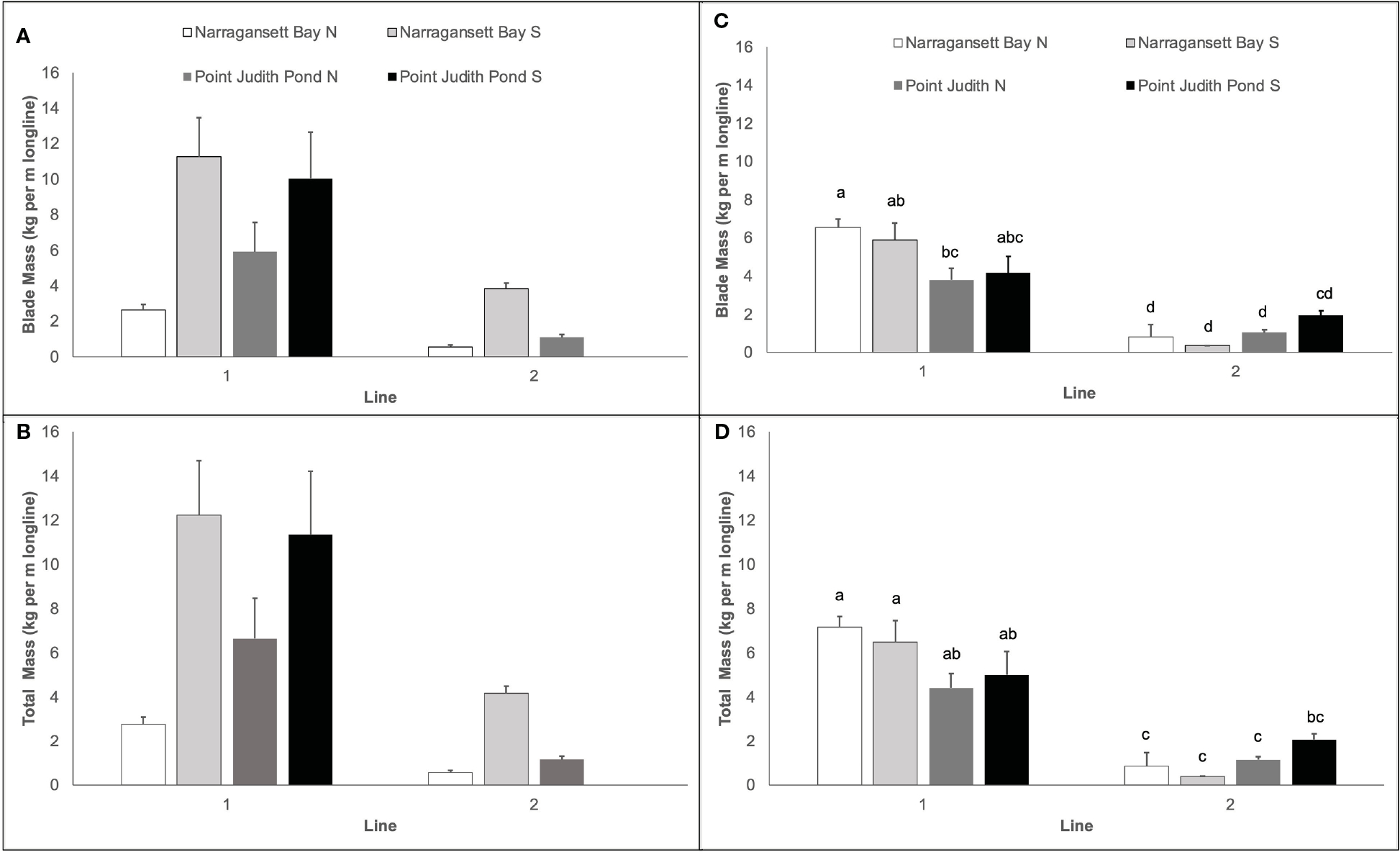
Kelp yield (kg per m of longline) from kelp blades in Year 1 **(A)**, total kelp (blades + stipe + holdfast) in Year 1 **(B)**, kelp blades in Year 2 **(C)**, and total kelp (blades + stipes + holdfasts) in Year 2 **(D)** at sites in Narragansett Bay and Point Judith Pond. Letters in panels (**C, D**) represent the results of *post hoc* analyses on significant interactions between site and line in Year 2; there were no significant interactions between site and line in Year 1 Panels **(A, B)**. Letters indicate the result of Tukey’s HSD post hoc comparisons (p<0.05); data points with a letter in common are not statistically different.

Kelp biomass yield varied by site and year and was always higher on Line 1. In Year 1, blade mass varied significantly among sites and lines (Table 1; Figure 6A) and was higher at Narr Bay S (7.55 kg per m of longline ± 1.93) than Pt. Judith N (3.49 kg per m of longline ± 1.31; p = 0.0098) and Narr Bay N (1.59 kg per m of longline ± 0.48; p = 0.0005). Total kelp (blade + stipe + holdfast) yield in Year 1 followed the same pattern (Table 1; Figure 6B). The highest total kelp yield was at Narr Bay S (8.21 kg per m of longline ± 2.11) and the lowest at Narr Bay N (1.67 kg per m of longline ± 0.52). Data from Pt. Judith S was not included in the Year 1 yield analyses due to missing data from Line 2 but had high blade (10.03 kg per m of longline ± 2.62) and total (11.33 kg per m of longline ± 2.89) kelp yield on Line 1 (Figures 6A, B).

In Year 2, kelp blade yield was highest at Narr Bay N (Line 1; 6.55 kg per m of longline ± 0.44) and lowest at Narr Bay S (Line 2; 0.36 kg per m of longline ± 0.01). Kelp blade yield was significantly higher on Line 1 at all sites except Pt. Judith S. Blade yield and total kelp (blade + stipe + holdfast) yield varied significantly by line and was dependent on site (Table 1; Figures 6C, D). Total kelp yield was highest at Narr Bay N (Line 1; 7.17 kg per m of longline ± 0.47) and lowest at Narr Bay S (Line 2; 0.37 kg per m of longline ± 0.01; Figure 6D).

### 3.6 Nutrient extraction

**FIGURE 7:**
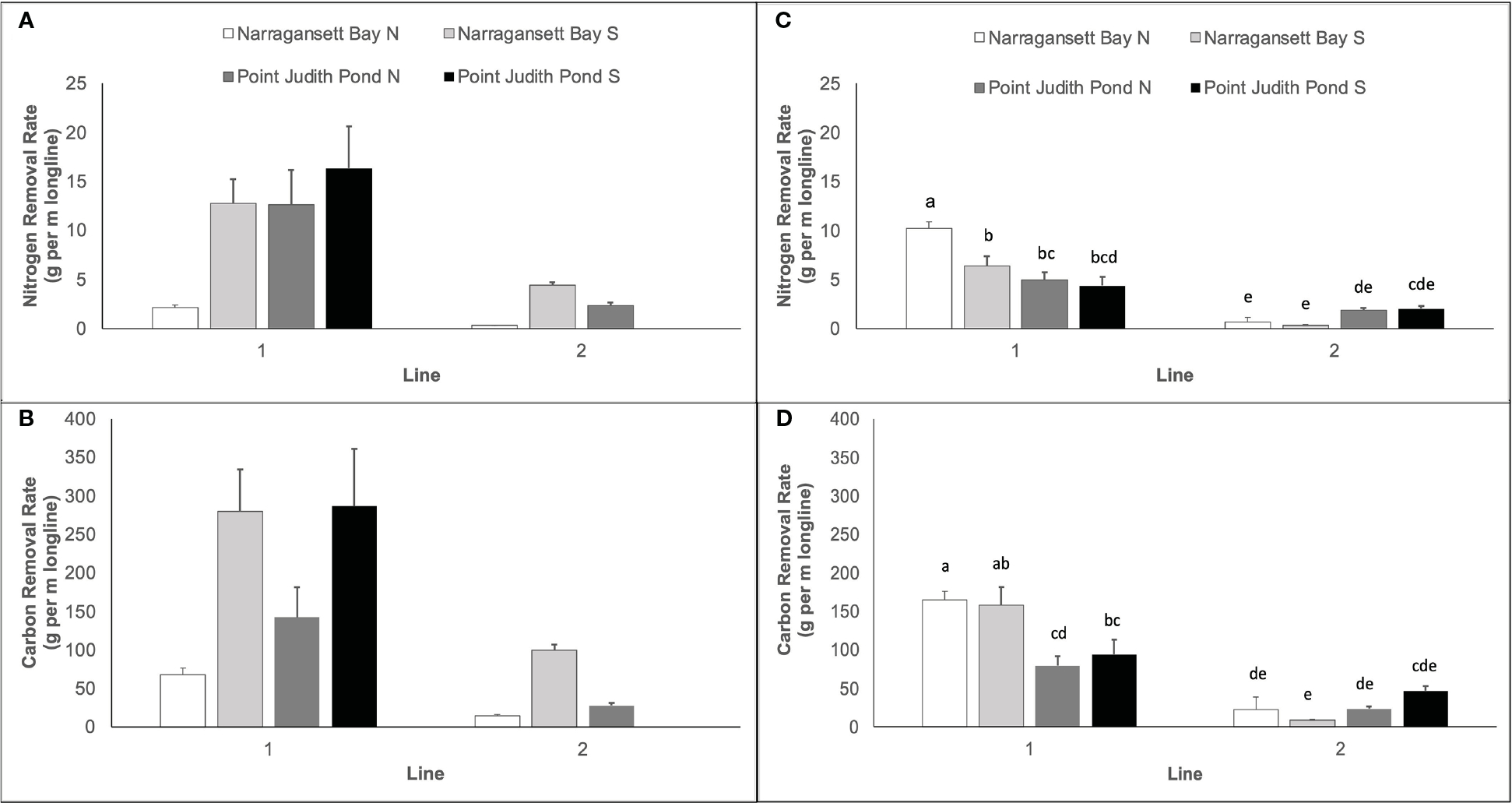
Nitrogen extraction (g per m longline) for Year 1 **(A)** and carbon extraction (g per m longline) for Year 1 **(B)**. Nitrogen extraction for Year 2 **(C)** and carbon extraction for Year 2 **(D)**. Each panel represents kelp blades cultivated at sites in Narragansett Bay and Point Judith Pond. Letters in panels **(C, D)** represent the results of *post hoc* analyses on significant interactions between site and line in Year 2; there were no significant interactions between site and line in Year 1 Panels **(A, B)**. Letters indicate the result of Tukey’s HSD post hoc comparisons (p<0.05); data points with a letter in common are not statistically different.

The mean calculated nitrogen extraction ranged from 0.28 ± 0.04 to 16.35 ± 4.26 g of N per m of longline (Figure 7). In Year 1, nitrogen and carbon extraction varied significantly among sites and lines (Table 1). The nitrogen extraction of kelp at Narr Bay S was significantly higher than Narr Bay N (8.55 ± 2.17 vs. 1.21 ± 0.43 g N per m of longline; p = 0.0036; Figure 7A); the carbon extraction of kelp at Narr Bay S (189.70 ± 47.21 g C per m of longline) was significantly higher than at both Narr Bay N and Pt. Judith N (41.32 ± 12.55 and 84.92 ± 31.16 g C per m of longline; p = 0.005 and 0.0071; Figure 7B). In Year 2, both nitrogen and carbon extraction of kelp varied by sites, but these effects were dependent on the kelp line (Table 1). For both Narr Bay N and Narr Bay S, the nitrogen (Figure 7C) and carbon (Figure 7D) extraction were significantly higher on Line 1 than on Line 2; there was no significant difference in the nitrogen and carbon extraction between lines at Pt. Judith S. Interestingly, the nitrogen and carbon extraction were highest at Pt. Judith Pond S in Year 1, but this site was amongst the lowest rates in Year 2.

### 3.7 Gross revenue

Based on upper reported market values ($3.30USD per kg; Engle et al., 2018) and the mass of kelp blades reported in this study, farmers growing kelp in Rhode Island have the potential to earn up to $2,229.48USD per 60 m of longline produced, though the average high-end value per longline was $1,089.33USD (Table 2). Using low end reported market values ($0.88 per kg; Piconi et al., 2020), farmers would earn a maximum of $594.53USD per longline, with an average earning of $290.49 (Table 2).

## 4 Discussion

### 4.1 Kelp performance and yield

This study represents one of the first attempts to grow kelp across different habitat areas at one time in southern New England and was based on the interest of oyster farmers who wish to diversify their crops. Kelp aquaculture has traditionally been conducted in waters deeper than 6 m in order to reduce the likelihood of kelp touching the bottom and reduce the risk of biofouling (Flavin et al., 2013). However, our data show that sugar kelp can be cultivated successfully during the winter months in both shallow coastal salt ponds and estuarine oyster farms in Rhode Island, USA. We found that both the timing of kelp deployment and placement of sites matter. The earlier in the fall that lines were put out into the field (once water temperatures are<15°C), the more kelp biomass was harvested during the following spring. Similarly, kelp aquaculture in Norway yielded larger thalli when lines were planted earlier (Matsson et al., 2019). The total yield of sugar kelp that we measured (6.65 – 12.24 kg/m long line) on Line 1 in Year 1 at all sites except Narr Bay N was comparable to other nearby regions including Long Island Sound, where Kim et al. (2015) reported sugar kelp yields of 5.5 – 9 kg/m long line. In the Gulf of Maine, where temperatures remain<15°C for longer than in southern New England, skinny kelp (*Saccharina angustissima* (Collins) Augyte, Yarish & Neefus) yields have been reported between 13.3 – 17 kg/m long line (Augyte et al., 2017); Umanzor et al. (2021) reported that yields of sugar kelp (2.5 kg/m long line) were similar to yields of skinny kelp at a site in New Hampshire, although this study had a truncated growing season due to permitting issues.

We found extensive variability in kelp blade length within and among different cultivation sites, and between years, which can create difficulties for farmers who need a standard sized product. Other kelp cultivation studies have noticed similar patterns; Grebe et al. (2021) found that trimming the end of kelp can be effective for maximizing kelp yield of *S. latissima* in Maine, depending upon the timing of trimming and the desired time of harvesting. Fine-scale genetic structure may also impact kelp blade growth rates and size; in the Gulf of Maine, considerable variation in genetic structure has been found across small spatial scales (Breton et al., 2018; Mao et al., 2020), although the impacts of fine-scale genotype differences on kelp frond phenotype are not well studied. In southern New England, a recent analysis reported that within-site genetic variability was higher than between-site variability for sugar kelp in southern New England (Mao et al., 2020). Here, all of our reproductive tissues were collected locally at one site, although we did not assess for population or individual-level differences in genetic structure. Lastly, biofouling can also create problems for kelp farmers (Marinho et al., 2015) by reducing tissue quality and consistency. Likely due to the timing of our harvest, we mostly avoided biofouling on the kelp fronds, which has been a considerable problem in other regions as it makes the blades unpalatable for human consumption (e.g., in Norway; Matsson et al., 2019). Biofouling on kelp in the northeastern United States generally increases after temperatures exceed 15°C in the spring; thus, the timing of harvest is critical to minimize crop loss due to biofouling (Flavin et al., 2013).

### 4.2 Tissue content and nutrient extraction

Kelp grown in Narragansett Bay showed a higher δ^15^N signature in nearly all cases than that grown in Point Judith Pond, reflecting well-established differences in nitrogen sources and processing between the two estuaries (Oczkowski et al., 2008). Although there were smaller differences in tissue nutrient quality (C:N) across sites and lines, the significant differences in nutrient extraction among sites and lines were driven primarily by large differences in frond biomass, not tissue quality. The very high C:N ratios at Narr Bay N in Year 1, Line 2 were driven by low %N and may explain the smaller blade size typically found at that site.

Our nitrogen and carbon extraction values were similar to previously reported rates from Long Island Sound by Kim et al. (2015) and in Danish waters by Marinho et al. (2015) and provide further evidence that kelp aquaculture can be used as a tool in bioremediation. Kim et al. (2015) reported nitrogen and carbon removal of ~5–20 g N/m and ~150–275 g C/m. Our nitrogen removal for Line 1 fell within this range, except at Narr Bay N in Year 2; our Line 2 values were always <5 g N/m. Our carbon extraction values for Line 1 fell within the range of Kim et al. (2015) except at Narr Bay N in Year 1 and at the Pt. Judith Sites in Year 2; our Line 2 values were always <150 g C/m. Marinho et al. (2015) reported *S. latissima* grown in Danish waters had N removal values of 0.5 – 7 g/m, consistent with the lower N extraction we observed on Line 2 in both years. However, dynamic energy budget modeling of this system has suggested that kelp growth is maximized when nutrients are available and competition is low (e.g., in the early spring); this is supported by the high C:N ratio in kelp from Narr Bay N in April of both years which indicates nutrient limitation or more competition from the spring phytoplankton bloom.

### 4.3 Diversifying sustainable aquaculture and site selection

The United States had a seafood trade deficit of US$17 billion and a per capita annual seafood consumption rate of 19 pounds in 2020 (National Marine Fisheries Service, 2022). This tremendous demand for seafood domestically cannot be met by wild capture fisheries and there is a need for sustainable aquaculture to expand considerably. Shellfish are a major aquaculture crop in the US, with oysters leading marine shellfish production by volume (42.3 million pounds) in 2019 (National Marine Fisheries Service, 2022). In the northeastern United States, oysters grow primarily during the summer months; here, we found that the growth rate of oysters at four aquaculture sites generally peaked in June-September and were positively correlated with the average water temperatures at three of the four sites. In contrast, kelp cultivation occurs during the winter months, making it a complementary crop to oysters. Adding kelp to existing oyster farms has the potential to diversify crops for aquaculture farmers, while requiring a low capital investment and providing an easier path to permitting (versus starting a new aquaculture farm). Additionally, seaweeds co-cultured with shellfish have a higher quality than those grown alone (e.g., Hargrave et al., 2022). Our results show that sugar kelp can be successfully incorporated into existing oyster farms in coastal salt ponds and in estuarine aquaculture sites in Rhode Island USA. Our results also indicate that kelp performance differs among sites, and that the characteristics of individual sites will dictate the level of success in kelp yield. At current market rates of US$0.88-$3.30 per kg, farmers in southern New England have the potential to earn US $2,229 per 60 m longline (Engle et al., 2018; Piconi et al., 2020).

Continued bottlenecks in the kelp cultivation industry, such as the ability of potential kelp farmers to identify a suitable site prior to beginning cultivation, have led to the development and incorporation of hydrodynamic and/or biodynamic models of kelp growth in specific regions. For example, Venolia et al. (2020) built a dynamic energy budget for *S. latissima* in aquaculture and found that the most important factors needed to accurately estimate kelp growth were temperature, irradiance, dissolved inorganic carbon, and dissolved inorganic nitrogen concentrations. Similarly, Broch et al. (2019) showed that the availability of nutrients and temperature differences across latitudes led to differences in predicted success cultivating *S. latissima* inshore and offshore in Norway. These models make it possible for farmers to measure the environmental conditions at sites and determine the potential for kelp growth prior to investing time and purchasing equipment for kelp farming.

### 4.4 Bottlenecks

While significant advancements in kelp cultivation have occurred (e.g., Peteiro et al., 2016; Hu et al., 2021), there are still several challenges that limit the ability of the kelp aquaculture industry to expand in the United States. These challenges include the availability of high-quality seed spools for farmers to plant on their farms, the lack of infrastructure to process kelp post-harvest, and the need for novel products that match market demands and make kelp aquaculture profitable for farmers (Engle et al., 2018; Kim et al., 2019). Additionally, climate change poses a risk to cold-adapted species such as kelps that are predicted to migrate poleward as water temperatures continue to rise (Gilson et al., 2021) and increasing temperatures have resulted in decreased kelp productivity in aquaculture (Hu et al., 2021). To ensure long-term resilience of these species, selective breeding programs focused on temperature-resistant strains of aquacultured species are needed (Kim et al., 2019; Bricknell et al., 2021; Hu et al., 2021). Shellfish species are also vulnerable to climate change, especially ocean acidification (Bricknell et al., 2021). The co-culture of seaweeds and shellfish may provide a way to alleviate the impacts of ocean acidification (Duarte et al., 2017; Xiao et al., 2021), though site conditions and cultivation seasons will strongly influence the success of this strategy.

### 4.5 Conclusions

Overall, our findings indicate that if an existing oyster farm is present in an environment where temperatures reliably drop below 15°C in the winter months, there may be lower barriers to farmers for adding on additional crops such as kelp, whether that farm is in a shallow coastal salt pond or a deeper coastal site. We also report variability in kelp performance and yield among sites and years, highlighting the need to understand site characteristics over time. Kelp farming in the United States relies on wild collected reproductive sporophytes to seed spools for planting, which has the advantage of potentially increased genetic diversity, but the downside of crop variability. Declining germplasm genetic diversity has been a major issue in established kelp aquaculture programs in Asia and has resulted in lower productivity and reduced blade quality (Barrento et al., 2016; Zhao et al., 2016; Hu et al., 2021). Successful incorporation of kelp onto existing shellfish farms has the potential to diversify crop production for farmers, while increasing the supply of sustainable aquaculture crops in the United States and providing important ecosystem services.

## Supplementary Material

Supplement1

## Figures and Tables

**TABLE 1:** Summary of p-values from statistical analyses.

	Year 1		Year 2	
	Line 1	Line 2	Line 1	Line 2
**Kelp blade length**	S:<0.0001* M:<0.0001* S × M: 00054*	S:<0.0001* M:<0.0001* S × M:<0.0001*	S: 0.0072 M:<0.0001* S × M:<0.0001*	S:<0.0001* M:<0.0001* S × M: 0.0001*
**Kelp blade width**	S:<0.0001* M:<0.0001* S × M: 0.6064	S:<0.0001* M:<0.0001* S × M: 0.2653	S:<0.0001* M:<0.0001* S × M:<0.0001*	S:<0.0001* M:<0.0001* S × M:<0.0001*
**Oyster shell height growth**			Measurements over 12 months (no lines) S:<0.0001* M:<0.0001* S × M:<0.0001*	
**Oyster shell length growth**			Measurements over 12 months (no lines) S: 0.0230* M:<0.0001* S × M:<0.0001*	
**δ^15^N**	S: 0.0065* M: 0.4895 S × M:0.4263	S:<0.0001* M: 0.0559 S × M: 0.9028	S:<0.0001* M:<0.0001* S × M: 0.1487	S:<0.0001* M:<0.0001* S × M:<0.0001*
**δ^13^C**	S:<0.0001* M:<0.0001* S × M:<0.0001*	S:<0.0001* M:<0.0001* S × M:<0.0001*	S:<0.0001* M:<0.0001* S × M:<0.0001*	S:<0.0001* M: 0.032 S × M:0.1647
**%N**	S:<0.0001* M:<0.0001* S × M: 0.0004*	S:<0.0001* M: 0.0007* S × M: 0.3324	S:<0.0001* M:<0.0001* S × M:<0.0001*	S:<0.0001* M: 0.0931 S × M: 0.9073
**%C**	S: 0.5871 M: 0.4089 S × M: 0.1606	S: 0.0187 M: 0.3117 S × M: 0.0164	S:<0.0001* M: 0.3811 S × M: 0.7934	S: 0.3028 M: 0.0379 S × M: 0.8458
**C:N**	S:<0.0001* M:<0.0001* S × M: 0.001*	S: 0.0003* M: 0.001* S × M: 0.0057*	S:<0.0001* M:<0.0001* S × M:<0.0001*	S:<0.0001* M: 0.0001* S × M: 0.2771
**Kelp blade yield**	S: 0.0006* L: 0.0002* S × L: 0.1015		S: 0.2064 L:<0.0001* S × L: 0.0099*	
**Total kelp yield**	S: 0.0008* L: 0.0003* S × L: 0.1074		S: 0.3031 L:<0.0001* S × L: 0.0224*	
**Nitrogen extraction**	S: 0.0027* L: 0.0005* S × L: 0.0803		S: 0.008* L:<0.0001* S × L: 0.001*	
**Carbon extraction**	S: 0.0005* L: 0.0003* S × L: 0.1163		S: 0.0426* L:<0.0001* S × L: 0.0019*	

S, Site; M, Month; S × M, Site × Month; L, Line; S × L, Site by Line.

Asterisks (*) indicate significance based on Bonferroni corrections for respective analyses.

**TABLE 2:** Gross revenue of kelp cultivation in Rhode Island based on final blade biomass from Line 1 at each site in Year 1 and Year 2.

Site	Year	Average Blade Mass at Harvest (kg per m)	Total Blade Mass on 60 m longline	Gross revenue per longline ($0.88USD per kg)	Gross revenue per longline ($3.30USD per kg)
**Narr Bay N**	Year 1	2.63	157.80	$138.86	$520.74
	Year 2	6.55	393	$345.84	$1,296.90
**Narr Bay S**	Year 1	11.26	675.60	$594.53	$2,229.48
	Year 2	5.89	353.66	$311.22	$1,167.08
**Pt. Judith N**	Year 1	5.90	354.30	$311.78	$1,169.18
	Year 2	3.80	228.04	$200.68	$752.55
**Pt. Judith S**	Year 1	N/A	N/A	N/A	N/A
	Year 2	4.17	250.39	$220.34	$826.29

Market values were derived from Piconi et al., 2020 ($0.88USD per kg) and Engle et al., 2018 ($3.30USD per kg). Data was not available for Pt. Judith S in Year 1. N/A, Not available.

## Data Availability

The original contributions and existing datasets presented in the study are hosted in Dryad and are publicly available. These data can be found here: Dryad https://doi.org/10.5061/dryad.hqbzkh1m3.
