# Supplementary material for: Integrated multi-trophic aquaculture with sugar kelp and oysters in a shallow coastal salt pond and open estuary site
Source: Front Aquac. Author manuscript; Available in PMC 2024 May 9. (PMC10581391; doi:10.3389/faquc.2023.1147524)
Supplement: Supplement1 [file NIHMS1933978-supplement-Supplement1.docx]

Supplementary Material

**Integrated multi-trophic aquaculture with sugar kelp and oysters in a shallow coastal salt pond and open estuary site**

Lindsay A. Green-Gavrielidis^*^, Carol S. Thornber, Autumn Oczkowski

*** Correspondence:** Corresponding Author: lgreengavrielidis@salve.edu

**Table S1.** Outplanting date, harvest date, and days cultivated for both kelp lines at each of the four sites in Year 1 and Year 2 of cultivation. Sites included Narragansett Bay North (Narr Bay N), Narragansett Bay South (Narr Bay S), Point Judith Pond N (Pt. Judith N), and Point Judith Pond S (Pt. Judith S).

| **Year** | **Site** | **Latitude and Longitude** | **Line #** | **Outplanting Date** | **Harvest Date** | **Days Cultivated** |
| --- | --- | --- | --- | --- | --- | --- |
| Year 1  2017-2018 | Narr Bay N | 41.575267  -71.437298 | 1 | 12/4/2017 | 4/21/2018 | 139 |
|  |  |  | 2 | 1/11/2018 | 4/21/2018 | 101 |
|  | Narr Bay S= | 41.544238  -71.420379 | 1 | 11/1/2017 | 4/21/2018 | 172 |
|  |  |  | 2 | 12/6/2017 | 4/21/2018 | 137 |
|  | Pt. Judith N | 41.406988  -71.502818 | 1 | 11/1/2017 | 4/17/2018 | 168 |
|  |  |  | 2 | 11/29/2017 | 4/17/2018 | 139 |
|  | Pt. Judith S | 41.402028  -71.507085 | 1 | 11/1/2017 | 4/22/2018 | 173 |
|  |  |  | 2 | 11/29/2017 | 4/22/2018 | 145 |
| Year 2  2018-2019 | Narr Bay N | 41.575267  -71.437298 | 1 | 12/19/2018 | 5/23/2019 | 156 |
|  |  |  | 2 | 2/11/2019 | 5/24/2019 | 103 |
|  | Narr Bay S | 41.544238  -71.420379 | 1 | 12/20/2018 | 5/24/2019 | 156 |
|  |  |  | 2 | 2/21/2019 | 5/24/2019 | 93 |
|  | Pt. Judith N | 41.406988  -71.502818 | 1 | 12/12/2018 | 5/3/2019 | 142 |
|  |  |  | 2 | 2/6/2019 | 5/3/2019 | 86 |
|  | Pt. Judith S | 41.402028  -71.507085 | 1 | 12/12/2018 | 5/3/2019 | 142 |
|  |  |  | 2 | 2/6/2019 | 5/3/2019 | 86 |

**Table S2.** List of exclusions/transformations for data analyses. Data were excluded from analysis of variance models when data were missing resulting in an unbalanced design. Data were transformed to meet the assumption or normality or equal variances where indicated. Sites included Narragansett Bay North (Narr Bay N), Narragansett Bay South (Narr Bay S), Point Judith Pond N (Pt. Judith N), and Point Judith Pond S (Pt. Judith S).

|  | **Year 1** | | **Year 2** | |
| --- | --- | --- | --- | --- |
|  | **Line 1** | **Line 2** | **Line 1** | **Line 2** |
| **Blade length** | *Data log 10 transformed* | Narr Bay N excluded  *Data log 10 transformed* | April excluded  *Data rank transformed* | April excluded  *Data log 10 transformed* |
| **Blade width** |  | Narr Bay N excluded  *Data log 10 transformed* | April excluded  *Data rank transformed* | April excluded  *Data log 10 transformed* |
| **Blade productivity** | Pt. Judith S excluded | Pt. Judith S excluded |  |  |
| **δ^15^N** | March excluded  *Data log 10 transformed* | January & Narr Bay N excluded | April excluded | April & Narr Bay N excluded |
| **δ^13^C** | March excluded | January & Narr Bay N excluded | April excluded | April & Narr Bay N excluded  *Data log 10 transformed* |
| **%N** | March excluded  *Data log 10 transformed* | January & Narr Bay N excluded | April excluded  *Data log 10 transformed* | April & Narr Bay N excluded |
| **%C** | March excluded  *Data log 10 transformed* | January & Narr Bay N excluded | April excluded | April & Narr Bay N excluded |
| **C:N** | March excluded | January & Narr Bay N excluded | April excluded  *Data rank transformed* | April & Narr Bay N excluded |
| **N and C extraction** | Pt. Judith S excluded | Pt. Judith S excluded |  |  |

**Table S3:** Environmental conditions measured at Narragansett Bay North monthly during the kelp cultivation season. nd=not detectable; two sampling points in May 2019 (5/2 and 5/23); measured at ~1 m depth.

| **Year** | **Month** | **PO_4_-P**  **(µM)** | **NO_3_ + NO_2_ (µM)** | **NH_4_ (µM)** | **DIN (µM)** | **Temp.**  **(°C)** | **Cond.**  **(mS/cm)** | **Salinity (psu)** | **pH** | **chl a (ug/L)** | **DO (mg/L)** |
| --- | --- | --- | --- | --- | --- | --- | --- | --- | --- | --- | --- |
| 1 | Nov. |  |  |  |  |  |  |  |  |  |  |
| 1 | Dec. | 0.63 | 7.59 | 1.60 | 9.19 | 8.28 | 47.41 | 30.55 | 7.85 | 7.2 | 10.04 |
| 1 | Jan. | 0.12 | 0.60 | 0.21 | 0.81 | -0.80 | 46.06 | 28.30 | 7.99 | 16.1 | 14.85 |
| 1 | Feb. | 0.10, 0.08 | 0.33, 7.32 | 0.17, 1.40 | 0.50, 8.72 | 2.52, 4.93 | 47.41, 42.97 | 29.80, 27.12 | 8.08, 7.68 | 27.7, 2.1 | 13.04 |
| 1 | March | 0 | 1.02 | 0.30 | 1.32 | 6.33 | 43.02 | 27.29 | 8.19 | 2.7 | 10.54 |
| 1 | April | 0.08 | 0.58 | 0.34 | 0.92 | 7.10 | 43.38 | 27.62 | 8.08 | 2.0 | 11.42 |
| 2 | Dec. | 0.71 | 10.07 | 1.36 | 11.43 | 5.72 | 45.55 | 28.99 | 8.01 | 2.8 | 15.57 |
| 2 | Jan. |  |  |  |  |  |  |  |  |  |  |
| 2 | Feb. | 0.23 | 2.64 | 1.79 | 4.43 | 3.09 | 46.19 | 29.11 | 8.15 | 3.2 | 12.87 |
| 2 | March | 0.13 | nd | nd | nd | 4.09 | 46.42 | 29.42 | 8.18 | 2.0 | 5.07 |
| 2 | April |  |  |  |  |  |  |  |  |  |  |
| 2 | May | 0.39 | nd | 1.43 | 1.43 | 14.5 | 44.72 | 28.98 | 7.98 | 2.8 | 7.97 |

**Table S4:** Environmental conditions measured at Narragansett Bay South monthly during the kelp cultivation season. nd=not detectable; two sampling points in May 2019 (5/2 and 5/23); measured at ~1 m depth

| **Year** | **Month** | **PO_4_-P**  **(µM)** | **NO_3_ + NO_2_ (µM)** | **NH_4_ (µM)** | **DIN (µM)** | **Temp.**  **(°C)** | **Cond.**  **(mS/cm)** | **Salinity (psu)** | **pH** | **chl a (ug/L)** | **DO (mg/L)** |
| --- | --- | --- | --- | --- | --- | --- | --- | --- | --- | --- | --- |
| 1 | Nov. | 0.81 | 2.04 | 0.61 | 2.65 | n/a | n/a | 31.00 | 7.8 | n/a | n/a |
| 1 | Dec. |  |  |  |  |  |  |  |  |  |  |
| 1 | Jan. | 0.06 | 0.67 | 0.17 | 0.85 | -0.70 | 47.95 | 29.59 | 8.06 | 19.4 | 13.99 |
| 1 | Feb. | 0.13, 0.09 | 0.66, 5.41 | 0.23, 2.14 | 0.89, 7.54 | 2.64, 5.14 | 47.49, 42.45 | 29.96, 26.78 | 8.13, 7.45 | 18.5, 2.1 | 11.73, 10.29 |
| 1 | March | 0.01 | 0.76 | 0.17 | 0.92 | 4.89 | 44.18 | 27.96 | 8.21 | 2.5 | 11.21 |
| 1 | April | 0.05 | 0.52 | 0.35 | 0.87 | 6.93 | 43.69 | 27.81 | 8.08 | 1.5 | 10.05 |
| 2 | Dec. | 0.81 | 8.57 | 1.64 | 10.21 | 6.36 | 46.08 | 29.44 | 7.95 | 3 | 14.14 |
| 2 | Jan. |  |  |  |  |  |  |  |  |  |  |
| 2 | Feb. | 0.26 | nd | 1.36 | 1.36 | 3.37 | 46 | 29.20 | 8.12 | 1.6 | 12.32 |
| 2 | March | 0.26 | nd | 1.29 | 1.29 | 3.89 | 46.5 | 29.44 | 8.17 | 1.7 | 10.49 |
| 2 | April |  |  |  |  |  |  |  |  |  |  |
| 2 | May | 0.42 | 1.07 | 1.32 | 2.39 | 13.53 | 45.71 | 29.61 | 7.97 | 2.7 | 8.12 |

**Table S5:** Environmental conditions measured at Point Judith Pond North monthly during the kelp cultivation season. nd=not detectable; two sampling points in May 2019 (5/2 and 5/23); measured at ~1 m depth.

| **Year** | **Month** | **PO_4_-P**  **(µM)** | **NO_3_ + NO_2_ (µM)** | **NH_4_ (µM)** | **DIN (µM)** | **Temp.**  **(°C)** | **Cond.**  **(mS/cm)** | **Salinity (psu)** | **pH** | **chl a (ug/L)** | **DO (mg/L)** |
| --- | --- | --- | --- | --- | --- | --- | --- | --- | --- | --- | --- |
| 1 | Nov. | 0.42, 0.09 | 6.11, 0.57 | 6.04, 0.20 | 12.15, 0.77 | n/a, 8.88 | n/a, 45.01 | n/a, 28.89 | 7.6, 7.9 | n/a, 9.6 | n/a, n/a |
| 1 | Dec. |  |  |  |  | 7.79 | 47.37 | 30.50 | 7.96 | 3 | 9.78 |
| 1 | Jan. | 0.02 | 1.18 | 0.20 | 1.38 | 3.86 | 45.21 | 28.67 | 7.81 | 25.9 | 11.14 |
| 1 | Feb. | 0 | 6.92 | 0.58 | 7.49 | 3.73 | 44.65 | 28.14 | 7.88 | 8.2 | 11.38 |
| 1 | March | 0.03 | 2.96 | 0.92 | 3.88 | 4.34 | 43.58 | 27.74 | 8.2 | 3.2 | 11.1 |
| 1 | April | 0.01, 0.01 | 4.12, 8.06 | 0.24, 0.59 | 4.36, 8.65 | 6.33, 9.06 | 43.02, 41.61 | 27.20, 26.52 | 8.15, 8.12 | 3, 5 | 11.45, 10.99 |
| 2 | Dec. | 1.07 | nd | 2.14 | 2.14 | 4.15 | 43.52 | 27.35 | 8.31 | 58.7 | 16.07 |
| 2 | Jan. |  |  |  |  |  |  |  |  |  |  |
| 2 | Feb. | 0.65 | 6.71 | 1.71 | 8.42 | 4.06 | 44.56 | 28.11 | 8.19 | 11.4 | 14.04 |
| 2 | March | 0.52 | nd | 1.29 | 1.29 | 5.67 | n/a | 27.56 | 8.26 | 5.8 | 12.1 |
| 2 | April | 0.16 | 1.43 | 1.14 | 2.57 | 11.41 | 44.33 | 28.55 | 8.19 | 3.3 | 5.58 |
| 2 | May | 0.19, 0.19 | 2.86, nd | nd, 1.14 | 2.86, 1.14 | 12.33, n/a | 40.07, n/a | 25.81, n/a | 8.15, n/a | 11.7, n/a | 9.5, n/a |

**Table S6:** Environmental conditions measured at Point Judith Pond South monthly during the kelp cultivation season. nd=not detectable; two sampling points in May 2019 (5/2 and 5/23); measured at ~1 m depth.

| **Year** | **Month** | **PO_4_-P**  **(µM)** | **NO_3_ + NO_2_ (µM)** | **NH_4_ (µM)** | **DIN (µM)** | **Temp.**  **(°C)** | **Cond.**  **(mS/cm)** | **Salinity (psu)** | **pH** | **chl a (ug/L)** | **DO (mg/L)** |
| --- | --- | --- | --- | --- | --- | --- | --- | --- | --- | --- | --- |
| 1 | Nov. | 0.49 | 3.83 | 5.10 | 8.93 |  |  |  | 7.6 |  |  |
| 1 | Dec. |  |  |  |  |  |  |  |  |  |  |
| 1 | Jan. | 0.04 | 0.45 | 0 | 0.45 | 3.94 | 47.04 | 29.79 | 7.93 | 12.2 | 11.35 |
| 1 | Feb. | 0 | 5.61 | 0.44 | 6.05 | 3.14 | 46.84 | 29.60 | 7.65 | 4.9 | 11.04 |
| 1 | March | 0.06 | 4.83 | 0.43 | 5.25 | 4.07 | 47.22 | 29.87 | 8.17 | 4.2 | 11.49 |
| 1 | April | 0.01 | 8.43 | 0.28 | 8.71 | 5.76 | 45.81 | 29.17 | 8.06 | 1.4 | 10.61 |
| 2 | Dec. | 0.36 | 1.50 | nd | 1.50 | 4.52 | 45.2 | 28.56 | 8.14 | 6.6 | 12.32 |
| 2 | Jan. |  |  |  |  |  |  |  |  |  |  |
| 2 | Feb. |  |  |  |  | 4.03 | 45.63 | 28.83 | 7.8 | 4.7 | 13.6 |
| 2 | March | 0.29 | nd | nd | nd | 5.45 | 44.2 | 28.02 | 8.36 | 11.1 | 13.75 |
| 2 | April | 0.23 | nd | 1.42 | 1.42 | 10.72 | 45.05 | 29.02 | 8.18 | 2.3 | 3.51 |
| 2 | May | 0.13, 0.26 | nd, nd | nd, 2.74 | nd, 2.74 | 10.58 | 44.99 | 28.98 | 8.07 | 8.2 | 8.51 |

**Figure S1.** Mean δ^15^N (in ‰) at the four farm sites from Year 1 showing Line 1 (planted in November, A) and Line 2 (planted in December, B) and Year 2 showing Line 1 (planted in December, C) and Line 2 (planted in January, D).

**Figure S2.** Mean δ^13^C (in ‰) at the four farm sites from Year 1 showing Line 1 (planted in November, A) and Line 2 (planted in December, B) and Year 2 showing Line 1 (planted in December, C) and Line 2 (planted in January, D).

**Figure S3.** Mean percent nitrogen at the four farm sites from Year 1 showing Line 1 (planted in November, A) and Line 2 (planted in December, B) and Year 2 showing Line 1 (planted in December, C) and Line 2 (planted in January, D).

**Figure S4.** Mean percent carbon at the four farm sites from Year 1 showing Line 1 (planted in November, A) and Line 2 (planted in December, B) and Year 2 showing Line 1 (planted in December, C) and Line 2 (planted in January, D).
